# Early detection efforts for colorectal and prostate cancer from the patient’s perspective over the course of 12 years: results of the KABOT survey study

**DOI:** 10.1017/S1463423624000653

**Published:** 2024-12-16

**Authors:** Kay-Patrick Braun, Julia Maurer, Ingmar Wolff, Torsten Vogel, Steffen Lebentrau, Matthias May, Markus Herrmann

**Affiliations:** 1 Institute of General Medicine, Otto-von-Guericke-University Magdeburg, Magdeburg, Germany; 2 MVZ Dr. Braun GmbH, Cottbus, Germany; 3 University Cancer Center UCC-R, University Hospital Regensburg, Regensburg, Germany; 4 Department of Urology, University Medicine Greifswald, Greifswald, Germany; 5 General Practice Torsten Vogel, Bernau, Germany; 6 Department of Urology, Otto-von-Guericke-University Magdeburg, Magdeburg, Germany; 7 Department of Urology, St. Elisabeth Hospital Straubing, Straubing, Germany

**Keywords:** General practitioner, family physician, colonoscopy, faecal occult blood test, PSA testing, cross-sectional study

## Abstract

**Aim:**

This study investigates the level of knowledge and utilization of colorectal cancer (CRC) and prostate cancer (PCa) early detection measures (EDMs) over a period of 12 years in general practice from the patient’s perspective.

**Background:**

The role of general practitioners (GPs) in EDMs for CRC and PCa in Germany is not well-documented with comprehensive data.

**Methods:**

We conducted a patient-centric survey in the German federal state of Berlin-Brandenburg at a 12-year interval to examine the role of GPs in EDMs for CRC and PCa. In 2009, 55 GPs were tasked with informing 50 consecutive male patients, each aged over 35, about participating in a survey study (study phase 1/SP1). To evaluate changes over 12 years, a new survey involving 50 male patients from each of 150 GPs was conducted from October 2021 to March 2022 (SP2).

**Findings:**

We thoroughly reviewed the questionnaires of 890 patients, with 755 in SP1 and 135 in SP2. Patients showed greater awareness of recommendations regarding colonoscopy compared to prostate-specific antigen (PSA) testing. GPs were the most frequently reported source of information for both EDMs in our cohort. Comparing the two study phases, no significant difference in specific awareness of colonoscopy or PSA testing was found among men eligible for EDMs. However, there was a notable increase in the role of health insurance companies as a source of information about colonoscopy over time. Nearly 60% of included patients underwent colonoscopy and/or PSA testing as EDMs.

**Conclusion:**

The number of EDMs performed among study participants did not increase over time. Our study confirms that GPs remain the primary source of information about EDMs among the study participants.

## 1. Introduction

Cancer remains a leading cause of mortality and a significant barrier to increased life expectancy worldwide (Sung *et al.*, 2021). This is partly due to the noticeable decline in mortality rates from stroke and coronary heart disease compared to cancer, a trend seen in many countries (Sung *et al.*, 2021). Colorectal cancer (CRC) and prostate cancer (PCa) are among the most prevalent malignancies affecting men globally: according to the epidemiological GLOBOCAN survey, 1,880,725 and 1,414,259 individuals were newly diagnosed with CRC and PCa, respectively, in 2020 (Sung *et al.*, 2021). In 2020, CRC and PCa were responsible for 9.2% and 3.8% of all cancer-related deaths worldwide (Sung *et al.*, 2021).

According to the 2019 German S3 ‘Guidelines for colorectal cancer’, CRC early detection (ED) should be recommended starting at the age of 50 years if an average risk of disease is present, while the guideline of the US Preventive Services Task Force recommend starting at 45 years (Leitlinienprogramm Onkologie/S3-Leitlinie Kolorektales Karzinom, 2019; US Preventive Task Force). Since 2019, the guidelines include a written invitation procedure for CRC screening (Richtlinien des Gemeinsamen Bundesausschusses). Colonoscopy represents the gold standard for diagnosing CRC due to its high sensitivity (95%) and specificity (90%) (Vogelaar *et al.*, 2006). Additionally, adenoma resection can be performed during this procedure, interrupting the adenoma-carcinoma sequence (Citarda *et al.*, 2001). Major risks of colonoscopy are significant bleeding, occurring in 14.6 out of 10,000 procedures, and perforation, occurring in 3.1 out of 10,000 procedures (Lin *et al.*, 2021). If an endoscopic procedure for ED is rejected, a stool test for occult blood can alternatively be performed. Compared to other stool tests, the immunochemical faecal occult blood test (iFOBT) achieves the best results (Nasir Kansestani *et al.*, 2022). Using the guaiac-based faecal occult blood test (gFOBT) as an example, several studies have found the crucial influence of the general practitioner (GP) on the frequency of cancer ED, with the primary care physician mostly educating about CRC and appropriate ED programmes and having the greatest influence on the ED behaviour of patients (Hadjipetrou *et al.*, 2017). However, relevant differences in knowledge about ED have been observed among this group of specialists (Sahin *et al.*, 2017). The patient perspective on early detection (ED) of CRC has, however, been insufficiently investigated to date – particularly studies from the years after 2020 are only sporadically available. Common among these studies is the finding that patients of the appropriate age for ED exhibit insufficient utilization of corresponding examinations, possess inadequate awareness, and generally favour stool-based ED tests over colonoscopy (Colon-Lopez *et al.*, 2023; Fayanju *et al.*, 2023; Yehia *et al.*, 2024; Zhu *et al.*, 2022).

Regarding PCa, all men over the age of 45 covered by statutory health insurances in Germany are exclusively entitled to a digital rectal examination (DRE). This examination is not an ED or screening procedure and is not suitable for the timely detection of PCa (Halpern *et al.*, 2018; Matsukawa *et al.*, 2024). In Germany, the determination of prostate-specific antigen (PSA) is performed exclusively at the patient’s request and after intensive information about the associated potential advantages and disadvantages (Leitlinienprogramm Onkologie/S3-Leitlinie Prostatakarzinom, 2021). Although the ultimate benefit of PSA-based screening has not yet been proven, the abolition of routine screening in the United States led to a significant shift towards locally advanced and metastatic tumour stages (Desai *et al.*, 2022). It is currently undisputed that PSA-based ED among men aged 50 to 65 years reduces PCa-specific mortality (CSM). Despite significant contamination in both study arms, the ERSPC trial unequivocally demonstrates a CSM reduction of 27% after 21 years (de Vos *et al.*, 2023). Potential harms of PSA-based ED programmes could arise from complications following a subsequent biopsy or from the consequences of overtreatment (Ilic *et al.*, 2013). The patient perspective on PCa early detection has also been inadequately studied. In the American PLACE project (*n* = 783 for questions on PCa early detection) and the Spanish PROSHADE study (*n* = 847), the majority of men of appropriate ED age reported having knowledge of PCa-specific ED. However, fewer than half of the participants were able to sufficiently answer general questions on the topic (Fayanju *et al.*, 2023; Parker *et al.*, 2024). Overall, it appears evident that even among men who have already undergone PCa-specific ED, there is better awareness of the benefits of ED than its disadvantages or harms (Braga *et al.*, 2020; Kuss *et al.*, 2021; Petrovic *et al.*, 2019; Walsh *et al.*, 2024).

Fundamentally, the GP plays a pivotal role as a gatekeeper in ED measures for CRC and PCa. Many patients initially consult only their GP and subsequently seek specialist physicians in other fields either as needed or upon referral by the GP. Thus, in this scenario, the GP is also responsible for overseeing occult blood control in faeces or determining the PSA level as part of PCa early detection. In Germany, GPs do not independently perform colonoscopies; therefore, a referral to a gastroenterologist is required in such cases. However, the German healthcare system, particularly for privately insured patients, offers the option to directly consult a specialist in gastroenterology or urology without a referral from the GP. The influence of these clinical pathways that patients can take in accessing oncological ED has not yet been systematically examined for CRC and PCa. The present study investigates whether attitudes towards ED behaviour have changed over the 12-year period and whether, for example, updated evidence and recommendations for PSA-based PCa ED are reflected in patient knowledge and behaviour. Additionally, the sources of information most important to the patients were examined. Patient knowledge concerning current ED tests is critical for decision-making. Since a written invitation programme by health insurers for CRC screening was introduced in Germany in 2019, this is of particular interest. We conducted a pilot study to serve as a basis for further investigations. In our literature search preceding this study, we could not find comparable long-term studies on patient knowledge and behaviour regarding CRC and PCa early detection in the German context. The only study that examined both patient awareness and willingness for early detection of CRC and PCa has highlighted differences in knowledge and behaviour regarding cancer ED, but not within a comparable regional and temporal framework (Fayanju *et al.*, 2023). The goal of this long-term study was to record patient perspectives on ED of these two commonly diagnosed cancers over a period of more than 10 years in a well-defined region of Germany and then compare them over time. In this context, our KABOT (‘Knowledge And Belief Over Time’) study represents a pioneering publication.

## 2. Methods

### 2.1. Implementation of the study

In 2009, an interdisciplinary working group comprising GPs, urologists, and gastroenterologists was formed to investigate the attitudes and knowledge of GPs and male patients regarding CRC PCa early detection in a cross-sectional study. A questionnaire with 20 items was developed, optimized, and validated through 12 structured individual interviews to assess comprehensibility.

During study phase 1 (SP1) in 2009, GP practices were identified in the German federal states of Berlin-Brandenburg. All physicians were assessed via a search of the respective Association of Statutory Health Insurance (SHI) Physicians using the search term ‘GP’, and 55 GPs were randomly selected. Each practice received 50 questionnaires for male patients who were at least 35 years old and able to complete them independently.

Study phase 2 (SP2) commenced in October 2021 with the aim of collecting longitudinal data. The study was labelled KABOT. SP2 was conducted in 150 GP practices in the federal state of Berlin-Brandenburg, surveyed similarly to SP1. For SP2, the surveys, which were otherwise identical to those in SP1, were expanded to include two additional thematic blocks. The 150 GP practices were selected by searching the homepages of the Brandenburg Association of Statutory Health Insurance (SHI) Physicians and the KV RegioMed teaching practices (search terms: GP, Cottbus, or Bernau, radius of 5 km) (www.kvbb.de). In January 2022, a reminder letter was sent to all initially contacted practices. All responses received by March 31, 2022, were included in the final analysis. Data collection took place exclusively in the practices of SHI-accredited GPs. Patients were invited to participate in the survey during their regular visits to the practice. Purely private practice physicians were excluded from the study. Surveys were conducted anonymously, and all data protection requirements were met.

Ethical approval for the study was granted by the Ethics Committee of the Brandenburg State Medical Association (2021-2126-BO-ff) on August 10, 2021, and the KABOT study was registered with the German Registry of Clinical Studies (DRKS registration number: 00027862) (www.drks.de).

### 2.2. Structure of the questionnaires

We adhered to the Consensus-Based Checklist for Reporting of Survey Studies (CROSS) in the development and analysis of the questionnaire (see Supplement 1) (Sharma *et al.*, 2021). The patient questionnaire for SP1 included 11 questions on general characteristics, family and vaccination history, and nine additional questions on knowledge, assessment, and personal status regarding participation in ED programmes (see Supplement 2). In SP2, the survey additionally included a question concerning the assessment of the impact of ED programmes and a block of questions on COVID-19 vaccinations (see Supplement 3). For both surveys, a pilot test was conducted with 12 patients each. Following the pilot test, adjustments were made to shorten the survey and ensure validity, clarity, plausibility, and completeness.

The response rate refers to those practices that indicated their participation by returning patient questionnaires.

### 2.3. Research questions and statistical methods

The main focus of the study was to determine whether and how patients’ knowledge of individual ED tests had changed over 12 years. The effective frequency of ED procedures for CRC and PCa, as well as changes over time, were investigated. Patients’ primary information channels about ED programmes were also assessed. In the case of incompletely answered questionnaires, available answers were nevertheless taken into account and appropriately reflected in the analysis.

In addition to the descriptive analysis of frequencies, univariate group comparisons were performed using the chi-square test. Data analysis was conducted with SPSS software (version 28.0; IBM Corp., Armonk, NY, USA). P-values were two-sided, and *p* < 0.05 was considered statistically significant for all tests.

## 3. Results

The composition of the study group based on individual evaluation criteria is shown in the flow chart in Supplement Figure 1. The questionnaires of 890 patients were thoroughly examined, with 755 in SP1 and 135 in SP2. The characteristics of the patients and the study group are presented in Table 1. 56.7% of the men participating in the study were between 55 and 74 years old, with no significant distribution differences between SP1 and SP2. Slightly more than one-third of the men (37.5%) had a university or college degree, with no significant difference between the two SPs. About 7 out of 10 participants were married at the time of the survey (SP1: 72.3%, SP2: 62.2%). Approximately half of the men were non-smokers who had never smoked (50.7%), with this proportion significantly increasing in SP2 compared to SP1 (63% vs. 48.5%). Nearly 92% of respondents were covered by statutory health insurance, with no significant differences over time.

**Table 1.** Overview of patient and study group characteristics

		**SP1 (** ***n*** **= 755)**		**SP2 (** ***n*** **= 135)**		**Total (** ***n*** **= 890)**	
		*n*	Percentage	*n*	Percentage	*n*	Percentage
**Age**	<35	29	3.8%	2	1.5%	31	3.5%
	35–39	38	5.0%	7	5.2%	45	5.1%
	40–44	43	5.7%	8	5.9%	51	5.7%
	45–49	61	8.1%	7	5.2%	68	7.6%
	50–54	86	11.4%	15	11.1%	101	11.3%
	55–59	120	15.9%	21	15.6%	141	15.8%
	60–64	70	9.3%	15	11.1%	85	9.6%
	65–69	126	16.7%	22	16.3%	148	16.6%
	70–74	117	15.5%	14	10.4%	131	14.7%
	From 75	64	8.5%	21	15.6%	85	9.6%
	Not specified	1	0.1%	3	2.2%	4	0.4%
**School education**	Primary school	199	26.4%	14	10.4%	213	23.9%
	Secondary school	276	36.6%	55	40.7%	331	37.2%
	High school	44	5.8%	8	5.9%	52	5.8%
	University	230	30.5%	52	38.5%	282	31.7%
	Not specified	6	0.8%	6	4.4%	12	1.3%
**Marital status**	Single	83	11.0%	28	20.7%	111	12.5%
	Married	546	72.3%	84	62.2%	630	70.8%
	Widowed	57	7.5%	7	5.2%	64	7.2%
	Divorced	68	9.0%	15	11.1%	83	9.3%
	Not specified	1	0.1%	1	0.7%	2	0.2%
**Children**	Yes	645	85.4%	108	80.0%	753	84.6%
	No	98	13.0%	24	17.8%	122	13.7%
	Not specified	12	1.6%	3	2.2%	15	1.7%
**Smokers**	Non-smoker	366	48.5%	85	63.0%	451	50.7%
	Former smoker	231	30.6%	18	13.3%	249	28.0%
	Smoker	155	20.5%	29	21.5%	184	20.7%
	Not specified	3	0.4%	3	2.2%	6	0.7%
**Insurance status**	Statutory	700	92.7%	118	87.4%	818	91.9%
	Private	50	6.6%	13	9.6%	63	7.1%
	Not specified	5	0.7%	4	3.0%	9	1.0%
**Flu vaccination**	Regular	431	57.1%	73	54.1%	504	56.6%
	Occasionally	160	21.2%	25	18.5%	185	20.8%
	Never	163	21.6%	36	26.7%	199	22.4%
	Not specified	1	0.1%	1	0.7%	2	0.2%

Overall, only 2.2% of patients had never heard of either ED test. 84.3% and 65.4% of the patients were aware of colonoscopy and PSA tests, respectively (*p* < 0.01). For both procedures, the GP’s practice was the most common source of information (58.3% vs. 46.9%, *p* < 0.01). Comparing the two study periods, in SP1, 84.8% of respondents had heard of colonoscopy, and 43.3% had already undergone the procedure. In SP2, the corresponding proportions were 81.5% and 54.1%, respectively. Regarding the PSA test, in SP1, 65.3% were aware of this ED test, and 42.9% had already undergone PSA testing. In SP2, these proportions were 65.9% and 52.6%, respectively.

The percentage of patients who actually underwent ED measures was statistically significantly higher in SP2 for both ED procedures when comparing the two survey periods (colonoscopy: *p* = 0.024, PSA: *p* = 0.039) (Table 2). A statistically significant increase in SP2 compared to SP1 was observed for the proportion of patients for whom health insurance was a source of information about colonoscopy (42.9% in SP1 vs. 52.6% in SP2, *p* = 0.046) (Table 2). The proportion of patients aged ≥50 years who were equally eligible for CRC screening in both SPs and had at least one of the recommended examinations (FOBT and/or colonoscopy) was 75.1% (438/583) in SP1 and 74.1% (80/108) in SP2. No statistically significant difference was observed over time (*p* = 0.810).

**Table 2.** Comparison of colonoscopy and PSA test

**Investigation** **procedure**	**SP1 (** ***n*** **= 755)**		**SP2 (** ***n*** **= 135)**		*p*
	*n*	Percentage	*n*	Percentage	
1. Which examination procedure have you heard of?					
**Colonoscopy**	640	84.8%	110	81.5%	0.368
**PSA test**	493	65.3%	89	65.9%	0.922
2. Which examination has already been carried out on you?					
**Colonoscopy**	327	43.3%	73	54.1%	0.024
**PSA test**	324	42.9%	71	52.6%	0.039
3. Which examination have you heard about from your family doctor?					
**Colonoscopy**	440	58.3%	79	58.5%	1.000
**PSA test**	349	46.2%	68	50.4%	0.400
4. Which examination have you heard of from your health insurance company?					
**Colonoscopy**	72	9.5%	21	15.6%	0.046
**PSA test**	47	6.2%	10	7.4%	0.569

In the subgroup analysis of patients of eligible age for screening/ED, no difference was found between SP1 and SP2 for either colonoscopy or PSA testing (Table 3). In the age group 50–64 years, 50.0% (138/276) in SP1 and 43.1% (22/51) in SP2 had already heard about PSA testing from their GP (*p* = 0.446). In this age group, it was performed in 44.2% (122/276) in SP1 and in 51.0% (26/51) in SP2 (*p* = 0.444).

**Table 3.** Comparison of colonoscopy, FOBT, and PSA testing among men eligible for early detection programmes (incl. reference to the respective eligible or recommended age groups)

**Investigation** **procedure**	**SP1**		**SP2**		*p*
	*n*	Percentage	*n*	Percentage	
**FOBT**					
Age limit	≥50 years		≥50 years		
Utilization	376/583	64.5%	65/108	60.2%	0.445
**Colonoscopy**					
Age limit	≥55 years		≥50 years		
Utilization	273/497	54.9%	65/108	60.2%	0.337
**PSA test**					
Age limit	≥45 years		≥45 years		
Utilization	321/644	49.8%	67/115	58.3%	0.105

A comparison of the frequency of colonoscopy and FOBT in patients aged <50 years is shown in Table 4. Additionally, 29.9% (266/890) of respondents underwent both examinations. Colonoscopy alone was performed in 15.1% (134/890) and PSA testing in 14.5% (129/890). Compared to the number of men who underwent either a colonoscopy or a PSA test, significantly more men had both examinations performed (*p* < 0.01). Further details are provided in Table 5.

**Table 4.** Frequencies of colonoscopy and FOBT performed in men under 50 years of age

**Investigation** **procedure**	**SP1 (** ***n*** **= 172)**		**SP2 (** ***n*** **= 27)**		
	*n*	Percentage	*n*	Percentage	*p*
**Colonoscopy**	29	16.9%	8	29.6%	0.118
**FOBT**	34	19.8%	8	29.6%	0.308

**Table 5.** Frequency of colonoscopy and PSA test

**Investigation procedure**	*n*	Percentage	*p*
**None**	361	40.6%	<0.01 (combination vs. single examination)
**PSA only**	129	14.5%	
**Colonoscopy only**	134	15.1%	
**PSA and colonoscopy**	266	29.9%	
**Total**	890	100.0%	

Of the 127 respondents in SP2 who reported having been vaccinated against COVID-19, 99 (78.0%) had also already had at least one screening test (iFOBT, colonoscopy and/or PSA assessment). In contrast, only three of the eight unvaccinated persons had undergone ED (37.5%, *p* = 0.021).

## 4. Discussion

Data on the frequency of utilization of screening colonoscopy in Germany show that since the introduction of colonoscopy screening for people with statutory health insurance in 2002, 7.14 million people had utilized this service by 2018. In 2018, a total of 445,052 individuals were found to have 73,466 (35.6%) adenomas and 3,758 carcinomas, of which 206,501 (46.4%) were men (ZI-Zentralinstitut, 2018). However, these numbers were not related to the number of individuals eligible for screening (ZI-Zentralinstitut, 2018). Data in the literature on the uptake of CRC screening range from 30 to 70% (Hultcrantz, 2021; Flander *et al.*, 2022). In the USA, uptake rates of up to 80% have been reported for colonoscopy (Hultcrantz, 2021). The frequency of CRC screening among eligible individuals in the present study was 75.1% in SP1 and 74.1% in SP2. However, an important finding is that no significant changes between the two study periods were observed.

The current consensus on CRC screening effectiveness is that it reduces mortality, but data from Finland do not support this claim (Pitkäniemi *et al.*, 2015; Bretthauer *et al.*, 2022). New methods of CRC screening, such as blood testing with Raman spectroscopy, have shown promising results in pilot studies (Jenkins *et al.*, 2023). It remains unclear whether these results can be confirmed in larger studies. An important finding of the current study is that, despite the rising CRC incidence in younger patients, neither the frequency of FOBT nor colonoscopy increased significantly in this age group over the 12-year observation period (GBD, 2022). However, it should be emphasized that this group is not adequately represented within our study cohort.

In Germany, no comparable figures are available for the actual use of PSA-based ED for PCa as described for CRC. Sieverding et al. determined that within the group studied in Germany, 48% of men had undergone PSA testing (Sieverding *et al.*, 2008). The frequencies of this testing procedure observed in our study among men eligible for ED programmes were 49.8% in SP1 and 58.3% in SP2, thus falling within these findings. Notably, the differences between the two SPs were not statistically significant. One explanation for the lack of increase could be the attitude of GPs towards PSA-based ED. In Germany, the proportion of GPs recommending PSA-based ED for asymptomatic men ranges from 51.2% to more than 80% (Lebentrau *et al.*, 2014; Kappen *et al.*, 2021). However, the same studies also found that 10.2% to 39.0% of GPs did not recommend PSA-based ED (Lebentrau *et al.*, 2014; Kappen *et al.*, 2019). Kappen et al. also reported that only 20.8% of GPs believed that PSA-based screening reduces PCa-specific mortality, whereas 74% of the GPs surveyed in Australia held this opinion (Ranasinghe *et al.*, 2015; Kappen *et al.*, 2021). Australian guidelines include a recommendation for PSA testing every 2 years for men aged 50-69 years after weighing the risks and benefits (PSA Testing).

Personal reasons for non-participation were another important factor why the number of ED procedures did not increase among men eligible for ED. Significantly more men had already undergone both colonoscopy and PSA testing compared to men who had undergone only one of the two examinations. Comparable results are also found in the literature (Carlos *et al.*, 2005). In the only study fundamentally comparable to ours in design, the US-based PLACE project, more than half of the men of appropriate age were unable to identify the correct age for each organ-specific ED (with 47.1% correctly identifying for PCa – 291/681 and 47.9% for CRC – 341/711) (Fayanju *et al.*, 2023). Consequently, there remains a significant need for specialized oncological education on ED, which should be primarily addressed by GPs and family doctors as the first point of contact and key facilitators for their patients. GPs must fulfil this responsibility by continually updating their specific oncological knowledge and adapting to new study findings and revised guideline recommendations (Atlas *et al.*, 2022, Estevan-Ortega *et al.*, 2024).

### 4.1. Strengths and limitations

The particular strength of the KABOT study lies in the long observation period of 12 years. This period includes changes to the recommendations on the starting age for preventive medical check-ups and the initiation of the health insurance companies’ invitation programmes. Comparable studies have not yet been conducted.

Some limitations must be considered when interpreting these results. Our questionnaire study is limited by sampling and selection biases. We consider this pilot study as descriptive and hypothesis-generating; further comprehensive studies on this topic should follow. The major limitation is the small number of participants in SP2, which is explained by the coincident timing with the coronavirus pandemic. Additionally, the age group of men below the screening age is underrepresented. Furthermore, there are differences in the characteristics of the participants between SP1 and SP2, particularly with regard to employment status. Another limitation of the present study is the possible selection bias. It remains unclear whether recruited participants were more likely to have an affinity for preventive measures. The fact that only a limited number of physicians provided questionnaires to their patients also represents a possible selection bias. It remains unclear whether the majority of physicians actively supports ED and provides information to their patients. Additionally, data collection was conducted exclusively in the practices of SHI-accredited physicians, which leads to a selection bias regarding the participant population. Another limitation of the study is that the questionnaire did not adequately capture patients’ knowledge about the potential risks of screening/ED. Therefore, data obtained in this study provide a starting point for future research. Further studies on attitudes towards ED behaviour are needed for a comparative evaluation of our results.

### 4.2. Clinical implications derivable from the study results as well as the presentation of further studies that now need to follow

The findings of our KABOT study suggest several clinical implications for improving patient care in the context of CRC and PCa early detection. First, GPs should continue to be the primary source of information for patients, as their role in educating patients is crucial for the success of ED programmes. To enhance this role, continuous professional development and training for GPs on the latest guidelines and benefits of ED measures are essential. Second, the observed increase in the influence of health insurers indicates that integrated approaches involving both healthcare providers and insurers could further promote awareness and uptake of screening programmes. Health insurers can play a pivotal role by sending reminders and providing educational materials about the importance of ED. Third, targeted interventions to increase awareness and participation in ED measures among men aged 50–64 years, particularly for PSA testing, are needed. This could include public health campaigns and community outreach programmes designed to address specific barriers and misconceptions about ED. Fourth, addressing the reasons for non-participation in ED measures, such as personal beliefs and lack of knowledge, is crucial. Tailored educational interventions that consider cultural and individual differences can help mitigate these barriers. Fifth, the study highlights the need for improved communication strategies to convey the potential benefits and risks (harms) of ED procedures to patients. Clear, understandable information can empower patients to make informed decisions about their health. Sixth, considering the rising incidence of CRC in younger populations, extending screening recommendations and promoting awareness of ED measures among individuals under 50 years old should be considered. This could involve updating current guidelines to reflect the changing epidemiology of CRC. Finally, further research is warranted to explore the impact of digital health technologies, such as telemedicine and electronic health records, in enhancing patient education and facilitating timely reminders for early detection measures. These technologies can bridge the gap between patients and healthcare providers, ensuring consistent and accessible information dissemination.

Further research is required to determine the extent to which screening/ED interventions can reduce cancer-related mortality and to conduct further risk-benefit analysis of these studies. This is important, taking into account the pivotal role of GPs as the source of information and the growing impact of health insurers. In particular, further studies should examine the extent to which the recently demonstrated benefit of PSA-based ED in terms of reducing mortality in the 50- to 65-year-old age group is reflected in patient knowledge and attitudes towards ED. This is of particular interest because no pertinent increase over time has been demonstrated in this age group in the present study. Although the incidence of CRC is increasing in the group under 50 years of age; this is not yet reflected in the attitudes towards colonoscopy or FOBT. This finding should stimulate further research even though this age group was underrepresented in this study.

## 5. Conclusions

Over the 12-year study period, GPs consistently remained the primary source of information for CRC and PCa early detection in our cohort. Even though this examination is performed exclusively in GP practices, it underscores the responsibility of this specialist group in educating patients about relevant examinations. To fulfil this responsibility, knowledge of associated benefits and risks is of utmost importance. Despite a significant increase in the rate of colonoscopy screenings for CRC over the 12-year period studied, overall screening behaviour (colonoscopy and/or FOBT) did not show a significant upward trend in men aged ≥50 years who were eligible for screening. This was also observed in the target group of men aged ≥45 years for PSA-based early detection of PCa. Furthermore, among men aged 50–64 years, who are of particular interest for early detection using PSA testing, no significant increase was observed between the two SPs – neither in terms of information provided by the GP nor in terms of the frequency of a previously performed test. Additionally, an increasing influence of health insurers as a source of information for patients on CRC screening was observed. This seems to reflect the initiation of the invitation procedure implemented since 2019.

## Supporting information

Braun et al. supplementary material 1 Braun et al. supplementary material

Braun et al. supplementary material 2 Braun et al. supplementary material

## Data Availability

The data supporting the findings of this manuscript are available upon request from the corresponding author. All relevant data and materials have been appropriately documented and will be provided promptly to interested researchers for the purpose of verification and further scientific inquiry. We are committed to transparency and fostering collaborative research efforts by facilitating access to our dataset.

## Supplementary material

The supplementary material for this article can be found at https://doi.org/10.1017/S1463423624000653.

## Clinical trial registration

German Registry of Clinical Studies (DRKS registration number: 00027862).
