# Supplementary material for: Early detection efforts for colorectal and prostate cancer from the patient’s perspective over the course of 12 years: results of the KABOT survey study
Source: Prim Health Care Res Dev. 2024 Dec 16;25:e68. doi: 10.1017/S1463423624000653 (PMC11669801; doi:10.1017/S1463423624000653)
Supplement: Braun et al. supplementary material 1 — Braun et al. supplementary material [file S1463423624000653sup001.docx]

**Supplement Table 1 (ST1)** Checklist for Reporting Of Survey Studies (CROSS)

| **Section/topic** | **Item** | **Item description** | **Reported on page #** |
| --- | --- | --- | --- |
| **Title and abstract** | | |  |
| Title and abstract | 1a | State the word “survey” along with a commonly used term in title or abstract to introduce the study’s design. | ST2 |
|  | 1b | Provide an informative summary in the abstract, covering background, objectives, methods, findings/results, interpretation/discussion, and conclusions. | ST2 |
| **Introduction** | | |  |
| Background | 2 | Provide a background about the rationale of study, what has been previously done, and why this survey is needed. | ST2 |
| Purpose/aim | 3 | Identify specific purposes, aims, goals, or objectives of the study. | ST2 |
| **Methods** | | |  |
| Study design | 4 | Specify the study design in the methods section with a commonly used term (e.g., cross-sectional or longitudinal). | ST2 |
|  | 5a | Describe the questionnaire (e.g., number of sections, number of questions, number and names of instruments used). | ST2 |
| Data collection methods | 5b | Describe all questionnaire instruments that were used in the survey to measure particular concepts. Report target population, reported validity and reliability information, scoring/classification procedure, and reference links (if any). | ST2 |
|  | 5c | Provide information on pretesting of the questionnaire, if performed (in the article or in an online supplement). Report the method of pretesting, number of times questionnaire was pre-tested, number and demographics of participants used for pretesting, and the level of similarity of demographics between pre-testing participants and sample population. | ST2 |
|  | 5d | Questionnaire if possible, should be fully provided (in the article, or as appendices or as an online supplement). | ST2 |
| Sample characteristics | 6a | Describe the study population (i.e., background, locations, eligibility criteria for participant inclusion in survey, exclusion criteria). | ST2 |
|  | 6b | Describe the sampling techniques used (e.g., single stage or multistage sampling, simple random sampling, stratified sampling, cluster sampling, convenience sampling). Specify the locations of sample participants whenever clustered sampling was applied. | ST2 |
|  | 6c | Provide information on sample size, along with details of sample size calculation. | ST2 |
|  | 6d | Describe how representative the sample is of the study population (or target population if possible), particularly for population-based surveys. | ST2 |
| Survey  administration | 7a | Provide information on modes of questionnaire administration, including the type and number of contacts, the location where the survey was conducted (e.g., outpatient room or by use of online tools, such as SurveyMonkey). | ST2 |
|  | 7b | Provide information of survey’s time frame, such as periods of recruitment, exposure, and follow-up days. | ST2 |
|  | 7c | Provide information on the entry process:  –>For non-web-based surveys, provide approaches to minimize human error in data entry.  –>For web-based surveys, provide approaches to prevent “multiple participation” of participants. | ST2 |
| Study preparation | 8 | Describe any preparation process before conducting the survey (e.g., interviewers’ training process, advertising the survey). | ST2 |
| Ethical considerations | 9a | Provide information on ethical approval for the survey if obtained, including informed consent, institutional review board [IRB] approval, Helsinki declaration, and good clinical practice [GCP] declaration (as appropriate). | ST2 |
|  | 9b | Provide information about survey anonymity and confidentiality and describe what mechanisms were used to protect unauthorized access. | ST2 |
| Statistical  analysis | 10a | Describe statistical methods and analytical approach. Report the statistical software that was used for data analysis. | ST2 |
|  | 10b | Report any modification of variables used in the analysis, along with reference (if available). | ST2 |
|  | 10c | Report details about how missing data was handled. Include rate of missing items, missing data mechanism (i.e., missing completely at random [MCAR], missing at random [MAR] or missing not at random [MNAR]) and methods used to deal with missing data (e.g., multiple imputation). | ST2 |
|  | 10d | State how non-response error was addressed. | ST2 |
|  | 10e | For longitudinal surveys, state how loss to follow-up was addressed. | ST2 |
|  | 10f | Indicate whether any methods such as weighting of items or propensity scores have been used to adjust for non-representativeness of the sample. | ST2 |
|  | 10g | Describe any sensitivity analysis conducted. | ST2 |
| **Results** | | |  |
| Respondent characteristics | 11a | Report numbers of individuals at each stage of the study. Consider using a flow diagram, if possible. | ST2 |
|  | 11b | Provide reasons for non-participation at each stage, if possible. | ST2 |
|  | 11c | Report response rate, present the definition of response rate or the formula used to calculate response rate. | ST2 |
|  | 11d | Provide information to define how unique visitors are determined. Report number of unique visitors along with relevant proportions (e.g., view proportion, participation proportion, completion proportion). | ST2 |
| Descriptive  results | 12 | Provide characteristics of study participants, as well as information on potential confounders and assessed outcomes. | ST2 |
| Main findings | 13a | Give unadjusted estimates and, if applicable, confounder-adjusted estimates along with 95% confidence intervals and p-values. | ST2 |
|  | 13b | For multivariable analysis, provide information on the model building process, model fit statistics, and model assumptions (as appropriate). | ST2 |
|  | 13c | Provide details about any sensitivity analysis performed. If there are considerable amount of missing data, report sensitivity analyses comparing the results of complete cases with that of the imputed dataset (if possible). | ST2 |
| **Discussion** | | |  |
| Limitations | 14 | Discuss the limitations of the study, considering sources of potential biases and imprecisions, such as non-representativeness of sample, study design, important uncontrolled confounders. | ST2 |
| Interpretations | 15 | Give a cautious overall interpretation of results, based on potential biases and imprecisions and suggest areas for future research. | ST2 |
| Generalizability | 16 | Discuss the external validity of the results. | ST2 |
| **Other sections** | | |  |
| Role of funding source | 17 | State whether any funding organization has had any roles in the survey’s design, implementation, and analysis. | ST2 |
| Conflict of interest | 18 | Declare any potential conflict of interest. | ST2 |
| Acknowledgements | 19 | Provide names of organizations/persons that are acknowledged along with their contribution to the research. | ST2 |

**Supplement Table 2 (ST2)** Representation of the location within the manuscript where all 19 items of the CROSS checklist were accounted for

| **Section/Topic** | **Item** | **Reported on page # or explanation for non-inclusion** |
| --- | --- | --- |
| ***Title and abstract*** | | |
| Title and abstract | 1a | 1 |
| Title and abstract | 1b | 2 |
| ***Introduction*** | | |
| Background | 2 | 4 |
| Purpose/Aim | 3 | 5 |
| ***Methods*** | | |
| Study design | 4 | 6 |
| Data collection methods | 5a | 7 |
| Data collection methods | 5b | 6 |
| Data collection methods | 5c | 6 and 7 |
| Data collection methods | 5d | Supplement 2 and 3 |
| Sample characteristics | 6a | 6 |
| Sample characteristics | 6b | 6 |
| Sample characteristics | 6c | The KABOT study fundamentally serves as a hypothesis-generating investigation, thereby lacking precedent studies from other research groups to validate a reliable effect size necessary for biometric sample size calculation. Furthermore, the study employed a highly structured form of cluster sampling in recruiting the study cohort, aiming to motivate the inclusion of approximately 50 patients from all general practitioners within clearly defined regions. This approach, too, posed challenges in conducting a biometric sample size calculation. |
| Sample characteristics | 6d | Throughout both study periods, a total of 29 general practitioners in the Berlin-Brandenburg region were motivated to participate, resulting in the distribution of 1450 questionnaires to patients. The response rate stood at 61.4% (890/1450), with rates of 72% during Study Period 1 (SP1) and 33.8% during SP2. The ethical approval for this study considered the return of questionnaires by the addressed patients as an expression of their intent to participate, thus separate informed consents were not mandated by the ethical approval. However, the decision by the Ethics Commission of the federal state of Brandenburg stated that non-respondent patients should not have descriptive criteria applied in the study, as their lack of response is to be construed as a refusal to participate. Consequently, we did not record data from the 560 non-respondent patients, thereby lacking the ability to demonstrate, in a comparison of descriptive criteria, how representative the participation rate of 61.4% is in reflecting the entirety of the addressed patients. |
| Survey administration | 7a | 6 |
| Survey administration | 7b | 6 |
| Survey administration | 7c | The transfer of the entire dataset from the returned questionnaires into the SPSS table used for statistical exploration was independently performed by two members of the research team (KPB and TV). Subsequently, various data entries were identified and cross-checked with patient information in the original questionnaires. This process significantly mitigated the presence of erroneous entries in the final SPSS table utilized for statistical analysis. |
| Study preparation | 8 | 6 and 7 |
| Ethical considerations | 9a | 7 |
| Ethical considerations | 9b | 7 |
| Statistical analysis | 10a | 8 |
| Statistical analysis | 10b | No modification of study variables was conducted. |
| Statistical analysis | 10c | 8 |
| Statistical analysis | 10d | 6 |
| Statistical analysis | 10e | For these analyses, this statement does not apply to the KABOT study. |
| Statistical analysis | 10f | For these analyses, this statement does not apply to the KABOT study. |
| Statistical analysis | 10g | Sensitivity analyses, in the strict sense, were not conducted in this analysis of the KABOT study. However, the validity of the presented results was examined concerning different age groups and also in relation to the patient's insurance status, which was an identified study objective. |
| ***Results*** | | |
| Respondent characteristics | 11a | 9 and 22 |
| Respondent characteristics | 11b | Due to the design of the KABOT study, there are two types of non-responders:  1.     The addressed general practitioners, of whom only 29 out of a total of 205 (14.2%) participated in the study despite reminders for their involvement. This is largely attributable to the insufficient density of general practices in the federal state of Brandenburg, where many general practitioners routinely work over 80 hours per week. The extensive workload simply leaves many colleagues with no spare time to support academic research. This observation was undoubtedly exacerbated during Study Phase 2 (SP2) due to the burdens of the COVID-19 pandemic. While 38.2% of the initially addressed general practitioners participated in SP1, this figure dropped to a mere 5.3% in SP2.  2.     The non-participation of 38.6% of male patients, on the other hand, falls within the expected range as known from other questionnaire-based studies involving patients. The reasons for this are diverse and have been analyzed in numerous studies on the subject. |
| Respondent characteristics | 11c | 7 |
| Respondent characteristics | 11d | The response to this item has been documented in Figure 1. Here's a brief summary: 1450 questionnaires were distributed by the 29 general practitioners to 1450 male patients, which were subsequently viewed by these patients. Ultimately, out of these initially 1450 patients, 890 patients (61.4%) opted to participate in the study and returned the completed questionnaires. |
| Descriptive results | 12 | Table 1 |
| Main findings | 13a | The descriptive analyses presented in the KABOT study were unadjusted. |
| Main findings | 13b | For the analyses presented in the KABOT study, this statement does not apply. |
| Main findings | 13c | The handling of non-respondent patients was previously addressed in our response to Item 6d, and the execution of subgroup analyses (as a substitute for sensitivity analyses) was detailed in our response to Item 10g. Among the 890 patients constituting the study group, some of the returned questionnaires were not fully completed for every question (the criterion for questionnaire inclusion was >95% completeness of responses). In Table 1, all missing data in individual questions were labeled as 'not specified,' with missing responses to individual study endpoints ranging between 1 and 12 (equivalent to 0.11% to 1.3%). |
| ***Discussion*** | | |
| Limitations | 14 | 12 |
| Interpretations | 15 | 12 and 13 |
| Generalizability | 16 | 11 and 12 |
| ***Other sections*** | | |
| Role of funding source | 17 | 16 |
| Conflict of interest | 18 | 16 |
| Acknowledgements | 19 | There are no additional doctors or institutions beyond those listed in the author group who require acknowledgment. A comprehensive acknowledgment was extended to the general practitioners participating over the 12-year study period, as well as to the patients. |

**Dear patients,**

**Preventive medical check-ups are a very important part of medical care. They are becoming increasingly important. By using preventive examinations, it is possible to detect and cure malignant tumour diseases in time. Cancer mortality can be reduced. However, regular use is a prerequisite. In order to obtain a statement about the frequency of the use of screening examinations and the level of information of the patients, we have decided to carry out a study. We are dependent on your cooperation in this.**

**We ask you to answer the following questions completely. Your information will be treated anonymously and used exclusively for scientific evaluation.**

**Thank you very much for your cooperation!**

**1. Personal details**

Age < 35 [ ] 35-39 [ ] 40-44 [ ] 45-49 [ ] 50-54 [ ] 55-59 [ ]

60-64 [ ] 65-69 [ ] 70-74 [ ] > 75 [ ]

School education Secondary school (8th grade) [ ]

Secondary school (10th grade) [ ]

Abitur [ ]

Study [ ]

Gainful employment Jobseeker [ ]

Employed [ ]

Independent [ ]

Pensioners [ ]

Marital status single [ ] married [ ] widowed [ ] divorced [ ]

Children yes [ ] no [ ]

Non-smoker [ ] former smoker [ ] Smoker [ ]

Insurance status statutory [ ] privately insured [ ]

Do you get regular flu vaccinations?

regularly [ ] occasionally [ ] never [ ]

When was your last tetanus (lockjaw) vaccination?

less than 5 years [ ] less than 10 years [ ] don't know [ ] I am not vaccinated [ ]

How often does a tetanus (lockjaw) vaccination have to be repeated?

annually [ ] every 5 years [ ] every 10 years [ ] does not have to be repeated [ ]

Was there or is there cancer in your family?

no [ ] one case [ ] several cases [ ]

When was your last preventive medical check-up?

I have never had [ ] for longer than 2 years [ ] within the last 2 years [ ]

**2. Several answers per line are possible to the following questions. The crosses refer to the examinations indicated in the table. Please also answer these questions completely!**

| **None** | **Blood test**  **in the stool** | **Blood test for prostate cancer**  **(PSA value)** | **Intestine-**  **mirror** | **Palpation of the rectum by the doctor** |
| --- | --- | --- | --- | --- |

1. Which of the above-mentioned studies have you heard of?

[ ] [ ] [ ] [ ] [ ]

Where did you hear about it ?

- Media (Newspaper,TV) [ ] [ ] [ ] [ ]

- from your family doctor [ ] [ ] [ ] [ ]

- Health insurance recommendation

[ ] [ ] [ ] [ ]

2. Which examinations do you feel sufficiently informed about?

[ ] [ ] [ ] [ ] [ ]

3. About which examinations do you feel you have **not** received sufficient information?

[ ] [ ] [ ] [ ] [ ]

4. Which of the above tests have you ever had?

[ ] [ ] [ ] [ ] [ ]

5. Which of the tests mentioned do you consider useful?

[ ] [ ] [ ] [ ] [ ]

6. Which of the above examinations do you plan to have carried out in the foreseeable future? [ ] [ ] [ ] [ ] [ ]

**Please only answer the following questions if you have already had one of the examinations.**

7. Have you ever had an abnormal result in one of the above-mentioned examinations?

[ ] [ ] [ ] [ ] [ ]

8. If, as asked under 7. a pathological result was once obtained. On the basis of the examination result of which examination were further steps taken?

[ ] [ ] [ ] [ ] [ ]

**Please hand in the completed questionnaire to your practice team!**

**Thank you very much for your cooperation!**

**Practice no. _**

**Dear patients,**

**Preventive medical check-ups are a very important part of medical care. They are becoming increasingly important. By using preventive examinations, it is possible to detect and cure malignant tumour diseases in time. Cancer mortality can be reduced. However, regular use is a prerequisite. In order to obtain a statement about the frequency of the use of screening examinations and the level of information of the patients, we have decided to carry out a study. We are dependent on your cooperation in this.**

**We ask you to answer the following questions completely. Your information will be treated anonymously and used exclusively for scientific evaluation.**

**Thank you very much for your cooperation!**

**1. Personal details**

Age < 35 [ ] 35-39 [ ] 40-44 [ ] 45-49 [ ] 50-54 [ ] 55-59 [ ]

60-64 [ ] 65-69 [ ] 70-74 [ ] > 75 [ ]

School education Secondary school (8th grade) [ ] Abitur [ ]

Secondary School (10th grade) [ ] Study [ ]

Gainful employment Jobseeker [ ] Employed [ ] Indipendend [ ] Pensioner [ ]

Marital status single [ ] married [ ] widowed [ ] divorced [ ]

Children yes [ ] no [ ]

Non-smoker [ ] former smoker [ ] Smoker [ ]

Insurance status statutory [ ] privately insured [ ]

Do you have regular flu vaccinations? regularly [ ] occasionally [ ] never [ ]

Have you been vaccinated against COVID-19 (Corona)? Yes [ ] No [ ]

What was the reason for the COVID-19 vaccination?

Meets at all Meets on

not too most too

Own wish to protect against the disease [ ] [ ] [ ] [ ] [ ]

Increasing prevalence of the 2G (recovered, vaccinated) rule [ ] [ ] [ ] [ ] [ ]

Recommendation of the general practitioner [ ] [ ] [ ] [ ] [ ]

more freedom to travel [ ] [ ] [ ] [ ] [ ]

When was your last tetanus (lockjaw) vaccination?

less than 5 years [ ] less than 10 years [ ] don't know [ ] I am not vaccinated [ ]

How often does a tetanus (lockjaw) vaccination have to be repeated?

annually [ ] every 5 years [ ] every 10 years [ ] does not have to be repeated [ ]

Has there been or are there any cases of cancer in your family? no [ ] one case [ ] several cases [ ]

When was your last preventive medical check-up?

I have never had [ ] for longer than 3 years [ ] within the last 3 years [ ]

**2. Several answers per line are possible to the following questions. The crosses refer to the examinations indicated in the table. Please also answer these questions completely!**

| **None** | **Blood test**  **in the stool** | **Blood test for prostate cancer**  **(PSA value)** | **Intestine-**  **mirror** | **Palpation of the rectum by the doctor** |
| --- | --- | --- | --- | --- |

1. Which of the above-mentioned studies have you heard of? [ ] [ ] [ ] [ ] [ ]

Where did you hear about it ?

- Media (newspaper, TV) [ ] [ ] [ ] [ ]

- from your family doctor [ ] [ ] [ ] [ ]

- Health insurance recommendation

[ ] [ ] [ ] [ ]

2. Which examinations do you feel sufficiently informed about?

[ ] [ ] [ ] [ ] [ ]

3. About which examinations do you feel you have **not** received sufficient information?

[ ] [ ] [ ] [ ] [ ]

4. Which of the above tests have you ever had?

[ ] [ ] [ ] [ ] [ ]

5. Which of the tests mentioned do you consider useful?

[ ] [ ] [ ] [ ] [ ]

6. Which of the above examinations do you plan to have carried out in the foreseeable future?

[ ] [ ] [ ] [ ] [ ]

7. Which of the above examinations can reduce cancer mortality?

[ ] [ ] [ ] [ ] [ ]

**Please only answer the following questions if you have already had one of the examinations.**

8. Have you ever had an abnormal result in one of the above-mentioned examinations?

[ ] [ ] [ ] [ ] [ ]

9. If a pathological result was obtained, as asked for under 8. On the basis of the results of which examination were further steps taken?

[ ] [ ] [ ] [ ] [ ]

**Please hand in the completed questionnaire to your practice team!**

**Thank you very much for your cooperation!**

**Practice no. _**
