# Supplementary material for: Early detection efforts for colorectal and prostate cancer from the patient’s perspective over the course of 12 years: results of the KABOT survey study
Source: Prim Health Care Res Dev. 2024 Dec 16;25:e68. doi: 10.1017/S1463423624000653 (PMC11669801; doi:10.1017/S1463423624000653)
Supplement: Braun et al. supplementary material 2 — Braun et al. supplementary material [file S1463423624000653sup002.docx]

Missing or not evaluable questionnaires

n=295

34 Practices without response

n=1700

Missing or not evaluable questionnaires

n=265

Total study group

n=890

evaluable questionnaires

n=135

8 participating practices

n=400

evaluable questionnaires

n=755

21 participating practices

n=1050

SP2

150 practices selected

n=7500

SP1

55 practices selected

n=2750

142 Practices without response

n=7100

***Figure 1:*** Flowchart showing the composition of the study group
